# Prevalence and association of oral manifestations with disease severity in patients diagnosed with COVID‑19: A cross‑sectional study

**DOI:** 10.3892/mi.2024.154

**Published:** 2024-04-10

**Authors:** Adriana-Teodora Campeanu, Mihaela Rus, Sorina Ispas, Alexandra Herlo, Aurora Craciun, Constantin Ionescu, Gheorghe-Mihail Banariu, Claudia-Simona Cambrea, Maria Nicolae, Andreea Nelson-Twakor, Irina-Magdalena Dumitru

**Affiliations:** 1 General Directorate of Social Assistance and Child Protection, 900178 Constanta, Romania; 2 Department of PhD Research, Faculty of General Medicine, ‘Ovidius’ University, 900470 Constanta, Romania; 3 Faculty of Law and Administrative Science, ‘Ovidius’ University, 900470 Constanta, Romania; 4 Department of Anatomy, Faculty of General Medicine, ‘Ovidius’ University, 900470 Constanta, Romania; 5 Department XIII, Discipline of Infectious Diseases, ‘Victor Babes’ University of Medicine and Pharmacy, 300041 Timisoara, Romania; 6 Department of Biochemistry, Faculty of General Medicine, ‘Ovidius’ University, 900470 Constanta, Romania; 7 Department of Infectious Diseases, Faculty of General Medicine, ‘Ovidius’ University, 900470 Constanta, Romania; 8 Infectious Diseases Clinical Hospital, 900178 Constanta, Romania; 9 Department of Pediatrics, County Clinical Emergency Hospital of Constanta, 900591 Constanta, Romania; 10 Department of Internal Medicine, County Clinical Emergency Hospital of Constanta, 900591 Constanta, Romania; 11 Faculty of General Medicine, ‘Ovidius’ University, 900470 Constanta, Romania; 12 Academy of Romanian Scientists, 050044 Bucharest, Romania

**Keywords:** oropharyngeal manifestations, oral cavity, COVID-19, pandemic, oral lesions

## Abstract

Systemic disorders may exhibit early signs when conducting an oral examination. Since the onset of the COVID-19 pandemic, several studies have been published detailing the direct impact of the virus on the oral cavity. The present study aimed to determine whether indeed there are any significant disparities in oropharyngeal manifestations between individuals infected with severe acute respiratory syndrome coronavirus 2 and a control group, and whether the virus has the ability to invade and reproduce inside oral keratinocytes and fibroblasts, resulting in the development of oral ulcerations and superficial lesions. The present study provides an overview of the symptoms that occur at an early stage of the illness, and the most commonly affected regions of the oral cavity, including the tongue, lips, palate and oropharynx are examined. In the present retrospective study, 52 patients infected with COVID-19 were recruited between April, 2021 and October, 2022. In addition, 52 individuals who tested negative for the virus were recruited as the control group. The study was conducted through a thorough examination and questionnaire provided to all participants. The results revealed that among the cohort of patients from the COVID-19 group examined (n=52), a proportion (mean, 16.15) displayed oral manifestations. Specifically, 75% of the patients in the COVID-19 group described oral cavity pain, and 69% of these patients had changes in teeth color or dental caries. In summary, in relation to the control group, the prevalence of oropharyngeal symptoms was generally lower compared to the COVID-19 group, apart from oral cavity pain (30.8%), tonsillitis (17.3%), bleeding (34.6%), teeth color changes (36.5%), recurrence (15.4%) and abscesses (7.7%). Thus, on the whole, the patients without COVID-19 had fewer oral manifestations.

## Introduction

The COVID-19 pandemic, which emerged in Wuhan, China, has presented a significant global public health crisis, affecting the majority of nations worldwide. The global effect has been significant due to its mechanisms of transmission, the subsequent development of severe acute respiratory syndrome, and the global mortality rate that followed the onset of the pandemic (1).

The World Health Organization (WHO) officially proclaimed a state of public health emergency on January 30, 2020(2). Subsequently, the virus has spread in >223 nations and regions, leading to a worldwide pandemic, with >700 million cases and almost 7 million deaths, as of October, 2023(3).

Laboratory genomic analysis demonstrated that the severe acute respiratory syndrome coronavirus 2 (SARS-CoV-2), which is responsible for COVID-19, shares a 96% overall genomic similarity with a bat coronavirus known as CoVZXC21 (RaTG13) (4). Additionally, there is a significant similarity of 80% sequence identity between this newly identified coronavirus and the SARS coronavirus (SARS-CoV-1), which caused the previous SARS pandemic in the past (5). The angiotensin-converting enzyme 2 (ACE-2) receptor in humans serves as the primary point of viral entrance into the human body. Notably, a single nucleotide substitution (Arg426 to Asn426) has been found to enhance the binding affinity of the new virus, perhaps accounting for its heightened transmissibility (6). SARS-CoV-2 mostly manifests as a respiratory virus (7). Consequently, the primary mode of transmission for this pathogen is the direct contact with Flügge's droplets from an individual infected that has symptoms, which are transmitted via coughing, sneezing or exhaling (8). The expression of ACE-2 receptors is higher in the small intestine, which may account for the presence of gastrointestinal symptoms observed in some of patients. There is a vast amount of evidence that supports the existence of viral genetic material in the stool of patients, hence establishing the fecal-oral pathway as a mechanism of transmission and spread of the virus (9).

The use of infection control measures is critical for controlling the transmission of the virus and for effectively managing the ongoing outbreak. The risk of infection between patients and dental practitioners may be greater in dental environments due to their specific features (10). Considering the probable impact of COVID-19 on dental practices and hospitals, there has been an urgent need for the implementation of rigorous and efficient infection control policies (11).

In combination with fever, fatigue, dry cough, myalgias, a sore throat, breathing difficulties and respiratory issues that can lead to severe acute respiratory syndrome, patients with COVID-19 may experience a diverse range of additional local and systemic complications (12). These include acute cardiac damage, acute renal failure, gastrointestinal complications, dysgeusia, anosmia and neurological symptoms (13).

The oropharyngeal microbiome, which is the community of organisms that colonizes the upper respiratory tract, can influence the clinical progression of respiratory viral infections, including SARS-CoV2, and the virus may be identified in saliva and oropharyngeal secretions (14). Aphthous-like ulcers and superficial necrosis were seen in individuals who had been diagnosed with COVID-19, as these lesions develop in areas that are recognized to possess ACE-2 receptors, such as tongue epithelium and salivary gland tissue, following the occurrence of dysgeusia (15).

## Patients and methods

In the present study examined the incidence of oropharyngeal manifestations in the context of SARS-CoV-2 infection. The differences between patients with COVID-19 group and a control group (uninfected group) were also examined as regards oral cavity symptoms.

### Characterization of the study groups

The present study was carried out between April, 2021 and October, 2022 at Constanța Clinical Hospital for Infectious Diseases (Constanța, Romania). The present study included two groups of patients and is part of a larger project associated with the doctoral thesis entitled: ‘Oropharyngeal manifestations in patients with compromised immunity’. The investigated groups were the following: i) a COVID-19 group, which included 52 patients diagnosed with SARS-CoV-2, admitted to the Constanța Clinical Hospital for Infectious Diseases; and ii) the control group, which consisted of 52 individuals that had appointments for different procedures and dental evaluations. These subjects tested negative with the nasopharyngeal swab that was performed on their appointment date.

The vaccination status of the individuals participating in the study was not part of the current research aims, as the present study aimed to document the oral cavity symptoms of those infected with the virus, without any oscillations that a vaccine would impose. Thus, data on whether the participants were vaccinated or not, and with which vaccine were not recorded.

The patients in the COVID-19 group underwent an objective examination of the oral cavity (both intraoral and extraoral), as well as paraclinical tests, i.e., laboratory analyzes which, in the case of some subjects, revealed the presence of fungi and/or bacterial infections, and radiological examinations to determine the severity of the condition.

Patients in both groups completed a questionnaire the purpose of which was to identify oropharyngeal manifestations before and during infection with SARS-CoV-2 for the COVID-19 group, in comparison with the control group. Patients were only included in the study if they had a confirmed infection and had oropharyngeal symptoms prior to the administration of cortisol-based therapy and the antiviral therapy. The questionnaire addressed the following: Demographic data, information related to oral hygiene, previous and current oropharyngeal manifestations and interaction with the dentist. The final section of the questionnaire addressed specific questions related to oral symptoms associated with COVID-19 and changes in taste and smell.

The present study received ethical approval from the Ethics Committee of Infectious Diseases Clinical Hospital in Constanta, Romania (protocol code 2 and date of approval February 24, 2021).

### Research hypotheses

The main aim of the present study was to identify the incidence of oropharyngeal manifestations in the context of SARS-CoV-2 infection, and to formulate specific research hypotheses. Thus, the following hypotheses were considered: i) The null hypothesis, where there are no significant statistical differences between the COVID-19 group and the control group as regards oropharyngeal manifestations. ii) Hypothesis 1, where statistically significant differences are anticipated regarding oropharyngeal manifestations between the group with COVID-19 and the control group. A higher incidence of oropharyngeal manifestations is assumed in subjects infected with SARS-COV-2.

### Statistical analysis

For the statistical analysis, data management and data visualization, the IBM Statistical Package for the Social Sciences Statistics 25 (SPSS 25; IBM Corp.) was used. To interpret the data, paired samples t-tests were performed, in which a one-sided P-value of 0.500 (in the YES paired-samples test) typically indicates that the observed data do not differ significantly from the null hypothesis. In statistical hypothesis testing, a P-value represents the probability of obtaining results as extreme or more extreme than the ones observed, assuming that the null hypothesis is true (16). A P-value of 0.500 suggests that the data analyzed do not provide strong evidence against the null hypothesis. In practical terms, this means that there is a lack of sufficient evidence to reject the null hypothesis in favor of hypothesis 1.

## Results

### Demographic characteristics of the subjects in the study groups

As shown in Table I, the age of the subjects varied from 23 to 88 years, with a mean age of 58 years in the COVID-19 group. In the control group, the age of the subjects ranged from 20 to 77 years, with a mean age of 47 years. The sex distribution in the COVID-19 group was 1/1, while the control group was mainly formed by females (77%). In both groups, the majority of the subjects were from urban areas (57.90% in the COVID-19 group and 56.88% in the control group). In the COVID-19 group, 28 (54%) subjects had completed secondary education and only 24 (46%) patients had a higher education, while in the control group, the majority of individuals had a higher education (69%).

### Incidence of oropharyngeal manifestations before the SARS-CoV-2 pandemic

The oropharyngeal manifestations that were analyzed in the group of subjects diagnosed with SARS-CoV-2, compared to the group of uninfected subjects at the time of data collection are presented in Table II.

Among the cohort of patients from the COVID-19 group examined in the present study (n=52), a proportion (mean, 16.15) displayed oral manifestations. Specifically, 75% of the patients in the COVID-19 group described oral cavity pain, and 69% of these patients had changes in teeth color or dental caries. A notable percentage of patients reported *Candida* (36.5%), herpes (40.4%), thrush (46.2%), tonsillitis (30.8%), pharyngitis (44.2%) and bleeding (34.6%). However, only 1.9% of patients in the COVID-19 group reported glossitis, and a small proportion of patients reported periodontal pockets (11.5%), abscesses (15.4%), other facial and oral lesions (23.1%) and recurrence of other oropharyngeal manifestations (13.5%) (Table II).

In the control group, the overall oropharyngeal manifestations were less when compared with those in the COVID-19 group, apart from glossitis (13.5%), tonsillitis (59.6%), pharyngitis (63.5%), bleeding (57.7%), periodontal pockets (25%) and abscesses (26.9%) (Table II).

The overall mean for the presence of oropharyngeal signs and symptoms for both groups was less when compared to the absence of these symptoms, with 16.15 (YES) vs. 35.85 (NO), and 20.31 (YES) vs. 32.38 (NO) for the COVID-19 group and CONTROL group, respectively (Table III).

Table IV presents the results of the paired samples t-test, which is a statistical procedure used to determine whether there is a significant difference between the means of two related groups (16). In numerous statistical tests, a common significance level (alpha) is set at 0.05. If the one-sided f is greater than alpha (i.e., P>0.05), it is typically considered non-significant, and it would fail to reject the null hypothesis (17). In this case, in the YES group, there was a P-value of 0.500, which suggests that the observed data do not exhibit a statistically significant deviation from the null hypothesis.

A P-value of 0.188 (in the NO paired samples test) is a numerical value that results from the statistical hypothesis test performed. In hypothesis testing, the P-value represents the probability of obtaining results as extreme or more extreme than the ones observed, assuming that the null hypothesis is true (18). In this case, a P-value of 0.188 suggests that, if the null hypothesis were true, there is an ~18.8% chance of observing the data or results that were obtained in the study. In the NO paired samples test, the one-sided P-value is greater than alpha (i.e., P>0.05) also, which means that the null-hypothesis cannot be rejected.

### Oropharyngeal manifestations in the COVID-19 group and in the control group during the pandemic

The findings of pathogens and symptoms of individuals recruited in the present study during the pandemic are presented in Table V. The results will be later compared with those before the outbreak. In the COVID-19 cohort, all patients presented periodontal pockets, a notable proportion of patients (40.4%) reported glossitis, while 38.5% had *Candida*. Some of participants indicated the presence of herpes (17.3%), thrush (21.2%), pharyngitis (30.8%), and bleeding (11.5%). On the other hand, a mere 1.9% of patients in the COVID-19 cohort indicated the presence of abscesses, while a very modest percentage of patients reported tonsillitis (7.7%), oral cavity pain (3.8%) and recurrence of oropharyngeal manifestations (5.8%).

As regards the control group, the prevalence of oropharyngeal symptoms was generally lower compared with that in the COVID-19 group, apart from oral cavity pain (30.8%), tonsillitis (17.3%), bleeding (34.6%), teeth color changes (36.5%), recurrence (15.4%) and abscesses (7.7%) (Table V).

As demonstrated in Table VI, within the sample of patients diagnosed with COVID-19, a fraction of these patients, with a mean value of 13, had oropharyngeal symptoms.

As aforementioned, a one-sided P-value of 0.500 generally suggests that there is no sufficient evidence to support the claim that the observed data deviate considerably from the null hypothesis (as shown in Table VII). The P-value in statistical hypothesis testing is a measure of the possibility of generating outcomes that are more likely than the observed data, under the assumption that the null hypothesis is valid (19). As shown in Table VII, a P-value of 0.500 was obtained for pairs, and this result indicates that the analyzed data do not provide substantial evidence to reject the null hypothesis. From a practical standpoint, it could be concluded that there is insufficient evidence to reject the null hypothesis in favor of an alternative hypothesis.

As aforementioned, in several statistical tests, it is expected to establish a significant threshold (alpha) of 0.05(16). If the P-value for a one-sided test exceeds the predetermined significance level (i.e., P>0.05), it is generally regarded as statistically non-significant. Consequently, the null hypothesis would not be rejected (18).

### Analysis of gustatory and olfactory alterations between the group of patients with COVID-19 and the control group

Of the 52 subjects in the COVID-19 group, 12 (23%) indicated an altered perception for spicy taste, while no subjects in the control group reported this change (Table VIII). A total of 13 (25%) patients reported a change in the perception of salty taste, compared to no subjects in the control group. In addition, 11 (21.2%) patients reported a change in the perception for sour taste, compared to no subjects in the control group, and 12 (23%) patients indicated an altered perception for sweet taste, while again, no subjects in the control group reported this change. A further 18 (34.6%) patients from the COVID-19 group reported changes in their sense of smell, compared to no subjects in the control group.

**Figure 1:**
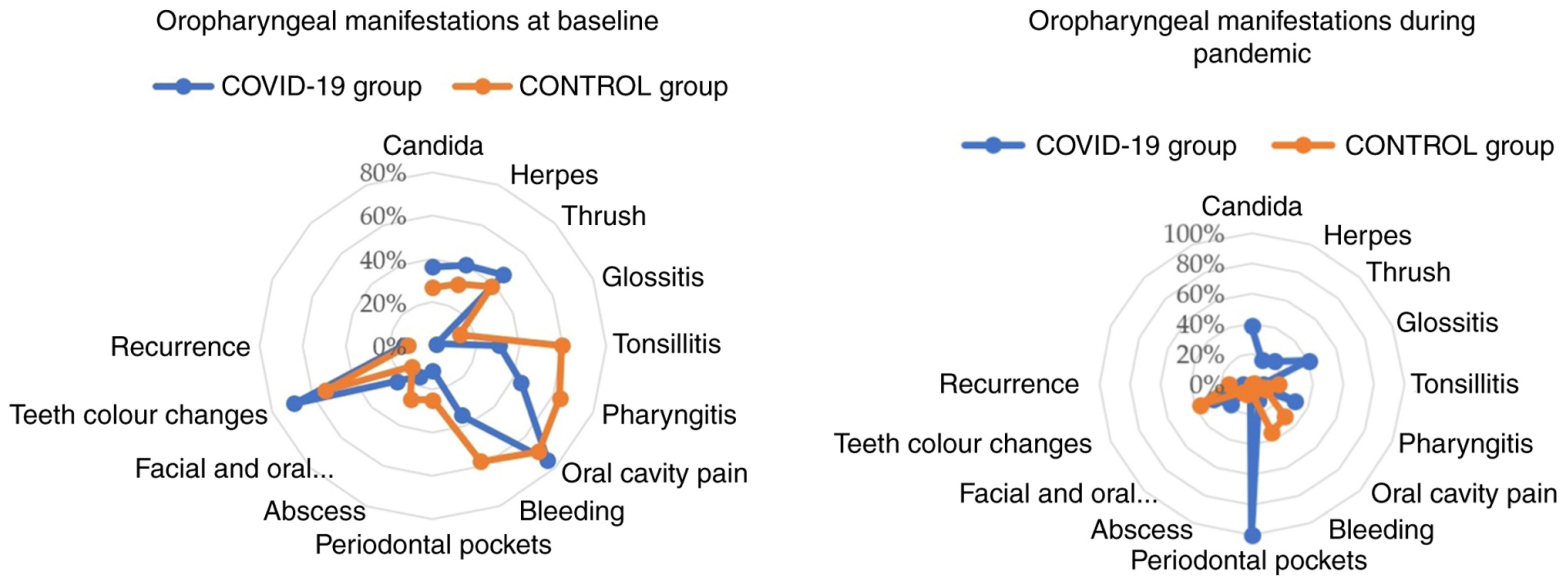
Comparison of the incidence of oropharyngeal manifestations between the two groups.

As demonstrated in Table IX, the mean number of patients that presented changes in taste and smell is lower when compared to that patients who did not experience these symptoms. In the present study, a decline in olfactory and gustatory abilities was observed among around a quarter of those diagnosed with COVID-19, in comparison to the ones that did not report these changes, and when it comes to the control group. Between 21 to 25% of patients reported experiencing either olfactory or gustatory dysfunction, as per their own accounts. The findings of the pathogens and symptoms of the individuals recruited in the present study before and during the pandemic are illustrated in Fig. 1.

Between 21 to 25% of patients reported experiencing either olfactory or gustatory dysfunction, and 34.6% reported loss of smell. As demonstrated in Table X, of the 52 patients with COVID-19 who were questioned and evaluated, 32 patients presented with severe forms of infection, 16 with moderate forms and only 4 patients presented with the mild form of COVID-19. Of these patients, 51 presented secondary diagnoses of which: 42 patients had respiratory failure, 23 patients had hypertension, 29 patients had liver diseases, 10 patients had diabetes mellitus. Of the 52 patients, 47 (90.4%) had cortisone-containing medications in their treatment regimen (Table XI).

**Figure 2:**
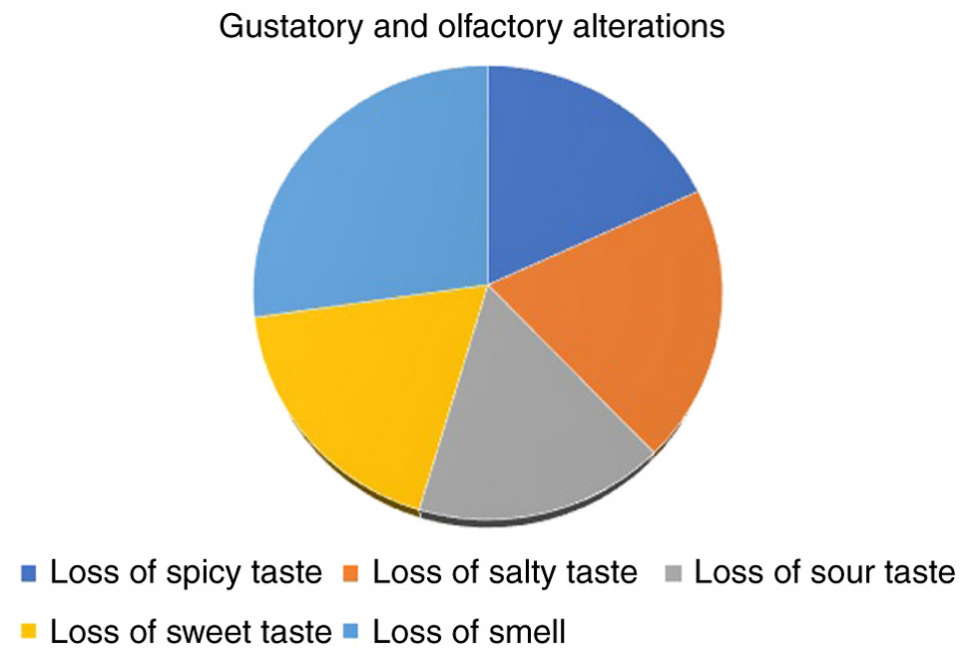
The findings of pathogens and symptoms of individuals recruited for the present study during the pandemic.

The gustatory and olfactory alterations of the patients in the COVID-19 group are illustrated in Fig. 2. It can be seen that the loss of sweet taste and loss of smell combined, comprise almost half of the gustatory and olfactory variations.

### Hypothesis testing: Forest plot

The hypothesis was examined using a paired samples t-test (Tables III and VI). Other statistical analyses were also conducted to test the hypotheses. A we forest plot was created, which is not typically used as a tool for directly testing hypotheses in the same manner that statistical tests, such as like t-tests, Chi-squared tests, or regression analyses are used (20). However, forest plots can indirectly inform hypothesis testing by providing a visual representation of the individual study results and the summary effect size (21). The forest plot also includes a summary effect size, often represented as a diamond. This summary effect size is calculated by pooling the results of all included studies (22). The null hypothesis in this case may be that the summary effect size is equal to zero or has no practical significance.

**Figure 3:**
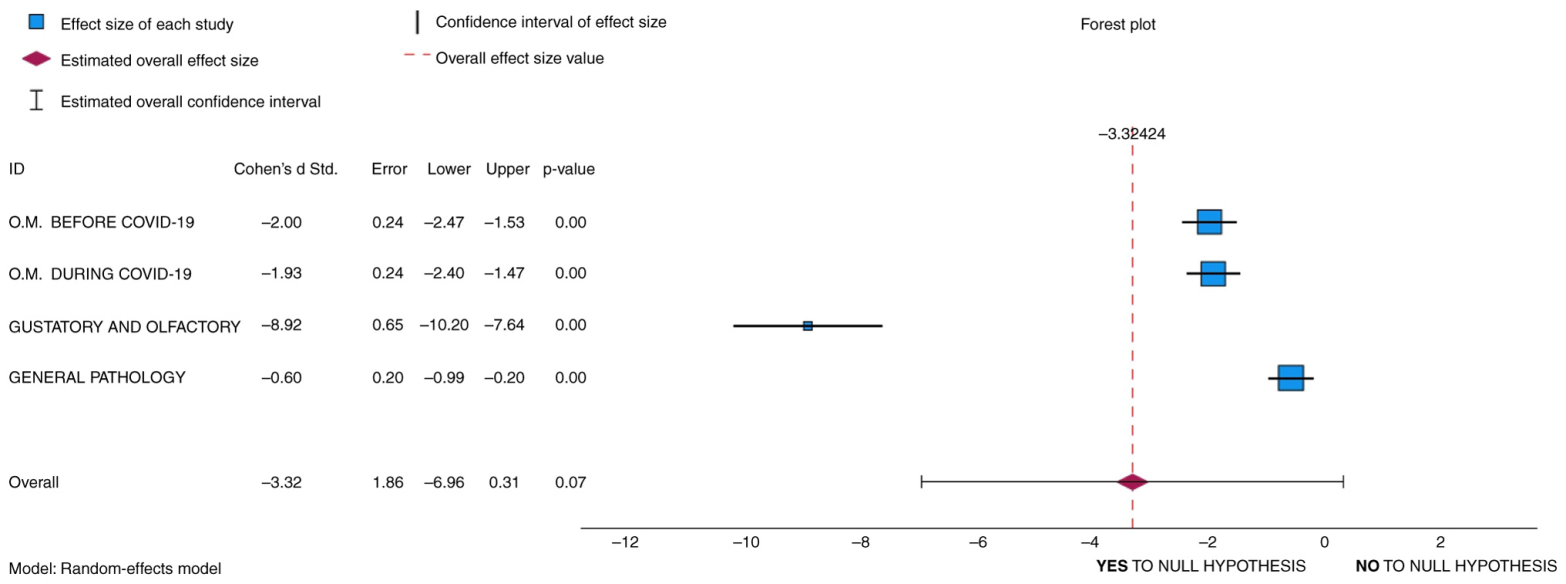
Forest plot created using the SPSS version 25 software. The variables taken into consideration are oropharyngeal manifestations before COVID-19, oropharyngeal manifestations after COVID-19, gustatory and olfactory changes and general pathology. The Y axis presents the variable's individual contribution on the overall effect (the middle red dotted line with a diamond shape) and where they are situated on the axis. The X axis presents the interval that confirms (YES) or denies (NO) the null hypothesis. The position of the red diamond on the X axis (overall effect) indicates if the variables taken into account prove and reject this hypothesis.

The forest plot included in Fig. 3 demonstrates a summary effect size, represented with a red diamond. Thus, if the summary effect size (the diamond) includes zero within its confidence interval, it can be concluded that there is no statistically significant effect, and the null hypothesis that there is no effect cannot be rejected (23). As shown in Fig. 3, although the diamond demonstrates an overall effect of -3.31, the 0 value is indeed within the confidence interval; thus, the null hypothesis cannot be rejected.

In addition, when Cohen's d is negative (i.e., in Fig. 3: -2, -1.93, -8.92, -0.6), it means that the COVID-19 group when compared to the control group has a lower mean or effect size. In other words, the observed effect goes in the opposite direction of what hypothesis 1 suggests, and that is a higher incidence of oropharyngeal manifestations is assumed in subjects infected with SARS-COV-2. After conducting this analysis, it was noted that a higher incidence of oropharyngeal manifestations is assumed in subjects infected with SARS-COV-2 compared to the control group.

An overall effect size of -3.31 suggests that, on average, the variables included in the analysis have a significant negative effect. However, since the overall effect is not exactly on 0, some statistical changes can be suggested in favor of hypothesis 1, although these are not sufficient to admit it as being correct. Since the null hypothesis often posits that there is no significant difference between groups or no effect of an intervention, and the result is not 0, it can then be concluded that, although the means of the COVID-19 and control groups are not equal, the null hypothesis can be accepted, as demonstrated.

### Cortisol and antiviral treatment

Corticosteroids are often used in the treatment of patients with COVID-19 with moderate to severe symptoms (24). Anosmia and olfactory symptoms in patients with COVID-19 are considered to be related to inflammation and damage to the olfactory nerves or receptors. These symptoms can vary in severity and duration among individuals (25).

The results of the present study demonstrated that 47 individuals (90.4%) in the COVID-19 group were receiving cortisone-based treatment (Table XI). Although corticosteroids may have an indirect impact on oropharyngeal symptoms by enhancing respiratory function, they are not specifically designed to address symptoms occurring in the throat or mouth (26).

The study by Richman and Nathanson (27) demonstrated that antiviral treatments are designed to target the replication and spread of the virus within the body. While they may aid in reducing the overall viral load and symptoms associated with COVID-19, the extent to which they can address specific symptoms, such as anosmia may vary (4). At present, anosmia, a condition characterized by the inability to perceive odors, mostly attributed to COVID-19 infection, may manifest as either partial or total and exhibit either transient or permanent effects (11,24). According to the study by Shamsundara and Jayalakshmi (25) the occurrence of anosmia is prevalent among the majority of individuals diagnosed with COVID-19, but often presents as a passing symptom. The same study mentioned that those identified with the virus had a much higher likelihood of experiencing olfactory dysfunction, with a 27-fold increase compared to the general population (25). In a randomized control trial, Rashid *et al* (26) concluded that symptoms, including ageusia were experienced together with anosmia in 234 individuals, accounting for 84.8% of the total participants of the study. The same study revealed that 83% of individuals had a complete resolution of anosmia during a period of 30 days (26). The median duration for recovery has been found to be 13 days (26). Hornuss *et al* (28) observed that 84% of individuals diagnosed with COVID-19 had either hyposmia or anosmia. By contrast, among the control group consisting of uninfected individuals, none of the participants reported anosmia, while 27% reported hyposmia (28).

However, it is important to note that anosmia can also arise from several other factors, such as allergies, the common cold and marked neurological impairments (4). The olfactory function serves as a defense mechanism for the human body against potential environmental dangers and pathogens (29). Several hypothesized causes of anosmia have been suggested, including the blockage of the olfactory cleft, inflammation in the nasal epithelium, the early death of olfactory cells, alterations in olfactory cilia, injury to the olfactory epithelium, and damage to the olfactory neurons or stem cell neurons (30).

## Discussion

The mode of transmission refers to the mechanism by which a disease or infection is spread from one individual to another (31). Based on the data derived from genetic and epidemiological studies, it is evident that the onset of the COVID-19 epidemic may be attributed to an initial instance of zoonotic transmission, subsequently leading to continuous human-to-human transmission (32). It is well known that the transmission of the virus mostly occurs via the upper respiratory tract (33). Furthermore, it is worth noting that there exists a potential for fecal-oral transmission, since scientific investigations have successfully detected the presence of SARS-CoV-2 in the feces of individuals originating from China and the USA (34).

A significant proportion of individuals had symptoms such as fever and dry cough, with a subset of patients also presenting with shortness of breath, exhaustion and other non-typical manifestations, including muscular pain, disorientation, headache, sore throat, diarrhea and vomiting (35). In a previous study, in a cohort of individuals who underwent a chest CT scan, the majority of patients had bilateral pneumonia, with the prevailing patterns being characterized by ground-glass opacity and bilateral patchy shadows (36).

A wide range of signs and symptoms have been linked to COVID-19, including dysgeusia and anosmia, even in cases when respiratory symptoms are not present. Olfactory and gustatory impairment are symptoms commonly observed in individuals diagnosed with COVID-19 and may serve as early indicators throughout the progression of the infection. The heightened knowledge of this fact has the potential to promote early diagnosis and treatment, as well as enhance vigilance in preventing viral transmission (37).

The present study demonstrated that 23% of the patients with COVID-19 had an altered perception for sweet and spicy taste, 25% experienced a change in the perception of salty taste, 21.2% reported a change in the sour taste, and no subjects in the control group reported any such changes. These results are in accordance with those from the study by Cattaneo *et al* (38), where ~45% of the patients in the COVID-19 group had symptoms concerning taste and smell, including the loss of olfactory or gustatory abilities. By contrast, none of the individuals in the control group reported experiencing these symptoms (38).

Salivary secretion is often compromised following infection with SARS-CoV-2, leading to the prevalent manifestation of xerostomia as the predominant oral symptom in individuals afflicted with COVID-19(39). The study conducted by Chen *et al* (39) demonstrated that xerostomia was present in >46% of the patients examined. They did not find any significant sex differences in the prevalence of xerostomia (39). Individuals diagnosed with xerostomia commonly exhibit a range of symptoms in addition to their primary complaint of oral dryness (40). These accompanying manifestations include a sensation of burning, altered taste perception (dysgeusia), inflammation at the corners of the mouth (angular stomatitis) and dysphagia (41). Although xerostomia is not fatal, it can significantly affect the quality of life and oral health of individuals (42).

As regards all the above, the present study revealed that in the COVID-19 group, between 30 to 44% of patients indicated tonsillitis, pharyngitis and oral cavity bleeding. On the other spectrum, a mere 1.9% of patients indicated the presence of glossitis, while ~11 to 15% reported periodontal pockets and abscesses. As regards the control group, the prevalence of oropharyngeal symptoms was generally lower compared to that in the COVID-19 group, apart from glossitis, tonsillitis, pharyngitis, hemorrhage, periodontal pockets and abscesses. These results are similar to the findings reported by other researchers, whose study revealed that glossitis was observed in both groups (positive and negative RT-PCR test groups) with similar relative frequencies (29).

It is worth noting that sialadenitis can also be observed in patients. In the study conducted by Fisher *et al* (43), the patient exhibited clinical manifestations indicative of concurrent acute bacterial suppurative parotitis and viral parotitis. Another study documented three instances of parotitis associated with COVID-19, and patients presented with unilateral ear pain and retromandibular edema, and magnetic resonance imaging revealed the presence of intracarotid lymphadenitis (44).

Parotitis refers to the inflammatory condition affecting the parotid glands, which are the most often affected major salivary glands (45). It has the potential to be either a localized condition or as a symptom of a broader systemic inflammation (46). Etiology may be attributed to several factors, including duct blockage (such as sialolithiasis), the presence of infectious agents, or inflammatory processes (Sjogren syndrome, rheumatoid arthritis, systemic lupus erythematosus) (47). Friedrich *et al* (48) found out that certain individuals diagnosed with COVID-19 have reported instances of salivary gland enlargement that affected the parotid glands. Even though the precise etiology of parotid gland enlargement in individuals with COVID-19 is still not fully clarified; nonetheless, it is suggested that this phenomenon may be associated with inflammatory processes, viral replication inside the salivary glands, or an immune-mediated reaction (49).

The present study demonstrated that infection was also associated with several skin facial and oral manifestations. Abscesses and other lesions accounted for 15 to 23% in the COVID-19 group and 13 to almost 27% in the control group. These slight differences between the two groups are in line with the findings reported in the literature. In their study, Nuno-Gonzalez *et al* (50) analyzed 666 patients over a 2-week period and concluded that 45% of them had various mucocutaneous manifestations. Of these patients, >25% had oral lesions, and swelling on the tongue was most commonly identified, followed by inflammation, redness of the tongue and canker sores. Several patients also reported a burning sensation in the mouth or a loss of taste. However, the oral symptoms proved to be temporary (50). These symptoms are not surprising, as it is common for viruses to cause both skin rashes and changes in the mucous membranes, such as ulcers or spots in the oral cavity (51).

According to experts, it is likely that these cases of tongue-COVID are not reported, as in some situations, doctors do not ask patients to open their mouths to examine their oral cavity, as such an examination increases the risk of infection. In addition, patients usually keep the mask on their face, as this protective measure is crucial to reduce the spread of the virus (52). In the present study, a reduction in olfactory and gustatory capacities individuals diagnosed with COVID-19, as compared to those who did not report such alterations. It was found that a considerable proportion of patients, ranging from 21 to 25%, indicated the presence of either olfactory or gustatory impairment. These findings are similar to another literature review that analyzed 10 studies describing olfactory dysfunction in a sample size of 1,627 individuals (53). That study (53) revealed a prevalence rate of >52% among patients diagnosed with COVID-19. In the same research, Tong *et al* (53) further examined nine studies to assess the occurrence of gustatory dysfunction, with a sample size of 1,390 individuals. This analysis also revealed a prevalence rate of almost 44% among the study group. The researchers performed subgroup analyses on studies that assessed olfactory dysfunction using both non-validated and validated instruments (53).

Further research led Amorim Dos Santos *et al* (54) to observe that ~21% of individuals diagnosed with COVID-19 had developed various oral mucosal lesions, which is a lower prevalence compared to dysgeusia and xerostomia. In that study, a significant proportion of individuals displayed oral mucosal lesions at ~10 days following infection. Subsequently, these patients commonly received treatment involving photo-biomodulation therapy and/or antiviral medication within a timeframe of 1 to 3 weeks (54). In addition to this conclusion, Iranmanesh *et al* (55) mentioned that individuals who are elderly, or those that have been hospitalized for an extended period of time, or exhibit poor hygiene practices, have diabetes, or are at an increased risk of developing oral mucosal lesions.

Likewise, these individuals often experience more severe, persistent and extensive oral lesions. These findings present a high prevalence of oral manifestations, including lesions such as aphthous, herpes, Kawasaki, plaque, fungal infections such as candidiasis and mucormycosis, mucosal petechiae, ulcers related to herpes simplex virus reactivation, oral herpes zoster, gingivitis and bleeding gums (56-59).

The present study had certain limitations which should be mentioned. The younger and more digitally engaged demographic has a greater propensity for social interaction, and this subgroup appears to be less susceptible to the impacts of COVID-19 in comparison to older cohorts, who have higher rates of illness and death (60).

Cross-sectional surveys may be susceptible to bias due to several factors, including the effect of confounding variables, variations in the timing of patient assessments in relation to their exposure, and potential reporting bias (61). In these investigations, such as the present study, the researchers are primarily presenting patient characteristics and their corresponding symptoms, rather than focusing on treatments, interventions, and their following impacts or outcomes (62).

This observational approach may contribute to the reduction of observer bias. When conducting research that relies on historical data obtained from patients, there exists a potential for memory bias and the under-reporting or errors of symptoms, particularly in relation to the timing and duration of symptoms (63).

In conclusion, impairment in olfactory and gustatory functions is often observed in individuals diagnosed with COVID-19, perhaps serving as early markers of disease progression. These manifestations were more frequent in the COVID-19 group, during SARS-CoV-2 infection compared to the control group. Compared to the control group, the COVID-19 group typically had a decreased prevalence of oropharyngeal symptoms, apart from oral cavity discomfort (30.8%), tonsillitis (17.3%), bleeding (34.6%), tooth colour changes (36.5%), recurrence (15.4%), and abscesses (7.7%). These symptoms were not associated with the severity of the disease, nor with the administration of cortisone therapy or antiviral therapy.

These manifestations may be an early sign of the disease and, taken into account, could lead to an early diagnosis, which would limit the transmission of infection in dental offices.

## Figures and Tables

**Table I:** Demographic characteristics of the patients in the COVID-19 and control groups.

	COVID-19 group				Control group			
Patient no.	Age, years	Sex^a^	Urban/rural^b^	Education^c^	Age, years	Sex^a^	Urban/rural^b^	Education^c^
1	65	2	1	1	20	1	1	1
2	65	2	1	2	20	1	1	2
3	35	2	1	1	25	2	2	2
4	37	1	1	2	26	2	1	2
5	42	2	1	2	27	2	1	2
6	63	1	1	2	28	1	1	1
7	62	2	2	1	33	2	1	2
8	72	1	2	1	35	1	1	2
9	75	1	1	1	35	2	1	2
10	71	1	1	1	36	2	1	1
11	81	1	1	2	37	1	1	2
12	59	1	2	1	38	2	1	1
13	71	2	1	1	39	1	1	2
14	52	2	1	2	40	2	1	1
15	59	1	1	2	40	2	1	2
16	61	1	1	1	41	2	1	2
17	46	2	2	2	43	2	1	1
18	51	2	1	1	43	2	1	2
19	64	2	1	1	44	2	1	2
20	55	1	1	1	44	2	1	2
21	68	2	1	1	44	2	1	2
22	83	1	1	2	44	2	1	2
23	77	1	1	1	44	2	2	2
24	68	2	1	1	44	2	2	2
25	54	1	1	2	45	2	1	2
26	49	2	2	2	46	2	1	2
27	24	1	1	1	47	2	1	2
28	74	2	1	2	47	2	1	2
29	54	2	1	2	48	1	1	2
30	88	2	1	1	49	1	2	1
31	53	1	1	1	50	2	1	2
32	23	1	1	2	51	2	1	2
33	70	2	1	1	51	2	1	2
34	60	2	1	1	51	2	1	2
35	29	2	1	2	52	1	1	1
36	55	2	1	2	52	2	1	2
37	45	1	1	2	53	1	1	2
38	75	1	1	1	53	2	1	2
39	48	2	1	1	53	2	1	2
40	33	1	1	2	55	2	1	1
41	61	2	1	1	56	2	2	2
42	56	2	1	2	59	2	2	1
43	67	1	1	1	60	2	1	1
44	54	2	1	2	60	2	1	2
45	36	1	1	1	62	2	1	2
46	64	2	1	2	70	2	1	1
47	58	1	1	2	72	2	1	1
48	71	1	1	1	74	1	1	1
49	62	1	1	2	74	1	1	2
50	65	2	1	2	74	2	1	2
51	64	1	1	1	75	2	1	1
52	63	1	1	1	77	2	1	1
Mean	58.4				47.81			
Total		26 (M)	47 (U)	28 (S)		12 (M)	46 (U)	6 (S)
		26 (F)	5 (R)	24 (H)		40 (F)	6 (R)	36 (H)
Variance	214.91				201.6			
Std. Dev.	14.66				14.19			

^a^Sex: 1, male (M); 2, female (F);

^b^urban/rural: 1, urban (U) residence; 2, rural (R) residence;

^c^education: 1, secondary level (S); 2, higher education level (H); 3, gymnasium level (G).

**Table II:** Oropharyngeal manifestations at baseline.

	COVID-19 group (n=52)		Control group (n=52)	
Pathogens and symptoms	No. of patients	%	No. of patients	%
Candida	19	36.5	14	26.9
Herpes	21	40.4	16	30.8
Thrush	24	46.2	20	38.5
Glossitis	1	1.9	7	13.5
Tonsillitis	16	30.8	31	59.6
Pharyngitis	23	44.2	33	63.5
Oral cavity pain	39	75.0	36	69.2
Bleeding	18	34.6	39	57.7
Periodontal pockets	6	11.5	13	25.0
Abscess	8	15.4	14	26.9
Facial and oral lesions	12	23.1	7	13.5
Teeth color changes	16	69.2	28	53.8
Recurrence	7	13.5	6	11.5

**Table III:** Statistical data for the oropharyngeal manifestations before the pandemic.

Group	Minimum	Maximum	Mean	Std. deviation
COVID-19 group				
Yes	1	39	16.15	9.856
No	13	51	35.85	9.856
Control group				
Yes	6	39	20.31	11.693
No	16	46	32.38	10.720

**Table IV:** Data from paired samples t-test: Before the COVID-19 pandemic.

			Significance	
Pairs	Test type	Difference in proportions	One-sided P-value	Two-sided P-value
Yes for COVID-19 and control groups	Mid-P-value adjusted binomial	0.001	0.500	1.000
No for COVID-19 and control groups	Mid-P-value adjusted binomial	-0.154	0.188	0.376

**Table V:** Oropharyngeal manifestations in the COVID-19 group and control group during the pandemic.

	COVID-19 group (n=52)		Control group (n=52)	
Pathogens and symptoms	No. of patients	%	No. of patients	%
Candida	20	38.5	0	0
Herpes	9	17.3	0	0
Thrush	11	21.2	0	0
Glossitis	21	40.4	1	1.9
Tonsillitis	4	7.7	9	17.3
Pharyngitis	16	30.8	5	9.6
Oral cavity pain	2	3.8	16	30.8
Bleeding	6	11.5	18	34.6
Periodontal pockets	52	100	3	5.8
Abscess	1	1.9	4	7.7
Facial and oral lesions	10	19.2	4	7.7
Teeth color changes	14	26.9	19	36.5
Recurrence	3	5.8	8	15.4

**Table VI:** Statistical data for oropharyngeal manifestations during the pandemic.

Group	Minimum	Maximum	Mean	Std. deviation
COVID-19 group				
Yes	1	52	13.00	13.441
No	0	51	39.00	13.441
Control group				
Yes	0	19	6.69	6.897
No	33	52	45.31	6.897

**Table VII:** Data from paired samples t-test: During the COVID-19 pandemic.

			Significance	
Pairs	Test type	Difference in proportions	One-sided P-value	Two-sided P-value
Yes for COVID-19 and control groups	Mid-P-value adjusted binomial	0.001	0.500	1.000
No for COVID-19 and control groups	Mid-P-value adjusted binomial	0.001	0.500	1.000

**Table VIII:** Gustatory and olfactory alterations observed in the present study.

	COVID-19 group (n=52)		Control group (n=52)	
Alteration	No. of patients	%	No. of patients	%
Loss of spicy taste	12	23	0	0
Loss of salty taste	13	25	0	0
Loss of sour taste	11	21.2	0	0
Loss of sweet taste	12	23	0	0
Loss of smell	18	34.6	0	0

**Table IX:** Statistical data for gustatory and olfactory alterations in the COVID-19 group.

COVID-19 group	Minimum	Maximum	Mean	Std. deviation
Yes	11	18	13.00	2.915
No	34	41	39.00	2.915

**Table X:** Severity of COVID-19 infection.

Severity	No. of patients	%
Severe form	32	61.5
Moderate form	16	30.8
Mild form	4	7.7
Total	52	100

**Table XI:** General pathological data of the patients in the COVID-19 group.

Variable	No. of patients	%
Secondary diagnosis		
Yes	51	98.1
No	1	1.9
Respiratory failure		
Yes	42	80.8
No	10	19.2
Hipertension		
Yes	23	44.2
No	29	55.8
Chronic renal disease		
Yes	6	11.5
No	46	88.5
Obesity		
Yes	8	15.4
No	44	84.6
Liver diseases		
Yes	29	55.8
No	23	44.2
Anxiety		
Yes	2	3.8
No	50	96.2
Autoimune diseases		
Yes	7	13.5
No	45	86.5
Cancers		
Yes	5	9.6
No	47	90.4
Diabetes		
Yes	10	19.2
No	42	80.8
Cortisone-based treatment		
Yes	47	90.4
No	5	9.6

## Data Availability

The datasets used and/or analyzed during the current study are available from the corresponding author on reasonable request.
